# Survey of the Effects of Exposure to 900 MHz Radiofrequency Radiation Emitted by a GSM Mobile Phone on the Pattern of Muscle Contractions in an Animal Model

**Published:** 2015-09-01

**Authors:** S. M. J. Mortazavi, S. Rahimi, A. Talebi, A. Soleimani, A. Rafati

**Affiliations:** 1 Ionizing and Non-ionizing Radiation Protection Research Center (INIRPRC), Shiraz University of Medical Sciences, Shiraz, Iran; 2 Medical Physics and Medical Engineering Department, School of Medicine, Shiraz University of Medical Sciences, Shiraz, Iran; 3 Department of Epidemiology, school of health, Tabriz university of medical science, Tabriz, Iran; 4 Physiology Department, School of Medicine, Shiraz University of Medical Sciences, Shiraz, Iran

**Keywords:** Non-Ionizing Radiation, Radiofrequency (RF), Electromagnetic Fields, GSM Mobile Phone, Muscle Contractions, Frog

## Abstract

**Background:** The rapid development of wireless telecommunication technologies over the past decades, has led to significant changes in the exposure of the general public to electromagnetic fields. Nowadays, people are continuously exposed to different sources of electromagnetic fields such as mobile phones, mobile base stations, cordless phones, Wi-Fi routers, and power lines. Therefore, the last decade witnessed a rapidly growing concern about the possible health effects of exposure to electromagnetic fields emitted by these sources.

**Materials and Methods:** In this study that was aimed at investigating the effects of exposure to radiofrequency (RF) radiation emitted by a GSM mobile phone on the pattern of contraction in frog’s isolated gastrocnemius muscle after stimulation with single square pulses of 1V (1 Hz), pulse height of contractions, the time interval between two subsequent contractions and the latency period were measured.

**Results:** Our findings showed that the pulse height of contractions muscle could be affected by the exposure to electromagnetic fields. Especially, the latency period was effectively altered in RF-exposed samples. However, none of the experiments could show an alteration in the time interval between two subsequent contractions after exposure to electromagnetic fields.

**Conclusion:** These findings support early reports which indicated a wide variety of non-thermal effects of electromagnetic radiation on amphibians including the effects on the pattern of muscle extractions.

## Introduction

Possible consequences of widespread application of microwave sources has caused a growing global concern. Many studies have been performed so far to detect possible effects of the exposure to microwave radiation on human nervous system and cognitive functions. The effect of electromagnetic field (EMF) created by common sources such as mobile phones or Wi-Fi routers on electrophysiological functions  is not clearly understood and current findings are controversial [1]. Substantial evidence indicates that mobile phone radiation affects brain activities. In 1998, Eulitz et al. showed that brain activity can be affected by radiations emitted from mobile phones when participants were processing task-relevant target stimuli. This effect could not be observed for irrelevant standard stimuli [2]. In the same year, Freude et al. reported that mobile phone radiation significantly affected preparatory slow brain potential in certain regions of the brain only when the subjects were performing a cognitive complex visual task. Interestingly, this effect could not be observed when subjects were perfoming a simple task [3]. On the other hand, Urban et al. in 1998 showed that five minutes of exposure to cell phone radiation could not cause significant alteration in visual evoked potentials [4]. Hladky et al. also reported that mobile phone use cannot change the visual evoked potential [5]. Later, Freude et al. confirmed their early findings that mobile phone radiation altered slow brain potentials when subjects were performing a complex task but they also showed that the irradiation could not significantly affect the participants in performing the behavioral task [6]. There is also a report by Jech et al. indicating that mobile phone radiation can change visual event-related potentials in narcolepsy patients performing a visual task [7]. Using PET scan, Aalta et al. in 2006 revealed a local decrease in regional cerebral blood flow under the antenna in the inferior temporal cortex. However, they reported an increase in the prefrontal cortex [8]. Hamblin et al. in 2004 showed that exposure to mobile phone radiation can lead to changes in event-related auditory evoked potential in participants when performing an auditory task. These researchers also showed an increase in reaction time in their participants, but no change was found in accuracy in the performance [9]. Other scientist have also reported different effects in the brain of exposed subjects during memory task [10, 11]. In 2005, Hamblin et al. confirmed that they were not able to replicate their earlier findings on auditory evoked potentials [9, 12].

In a study aimed at testing excitability of each brain hemisphere by transcranial magnetic stimulation, Ferreri et al. reported that after 45 minutes of exposure to mobile phone radiation, intracortical excitability was significantly altered with a decreased inhibition and enhancement in facilitation [13]. Papageorgiou et al. in 2006 reported that changes in P50 evoked potential indicated that exposure to mobile phone radiation altered pre-attentive working memory information processing [14]. On the other hand, Yuasa et al. reported that 30 minutes of exposure to mobile phone radiation could not significantly alter the human somatosensory evoked potentials [15]. Over the past years, our laboratory has focused on studying the health effects of exposure of laboratory animals and humans to some common and/or occupational sources of electromagnetic fields such as mobile phones [16-23] and their base stations [24], mobile phone jammers [25], laptop computers [26], radars [17], dentistry cavitrons [27] and MRI [28, 29].  This study is aimed at investigating the effects of exposure to radiofrequency radiation emitted by a GSM mobile phone on the pulse height of contractions, the time interval between two subsequent contractions and the latency period of frog’s isolated gastrocnemius muscle after stimulation with single square pulses of 1V (1 Hz).

## Material and Methods

### Animals

Frogs of both sexes (20-30 g) were obtained from Animal Lab of the Physiology Department, SUMS. Animals were kept in plastic containers in a room (20 ± 1°C) for one week before the experiments. The tap water in the plastic containers was changed 2 times a week.

### Exposure

Control frogs were kept in special cages during the sham exposure phases, while mobile phone group was exposed to 900 MHz radiofrequency radiation emitted from a common cellular phone (Nokia 1616) at a distance of 15 cm from the receiver for 30 minutes. Isolated gastrocnemius muscles, in the next stage, were exposed to switched on/off mobile phone radiation for 3 subsequent 10 minute intervals.

### Experiment Setup

Frogs were double pithed using a needle. Then the skin over the gastrocnemius muscle and its distal tendon was removed. Isolated gastrocnemius muscles were attached to the force transducer with a string. Nerve and muscle stimulations were performed separately. Using a PowerLab device (26-T), the pattern of muscular contractions was monitored after applying single square pulses of 1V (1 Hz) as stimuli. The pulse height of contractions, the time interval between two subsequent contractions and the delta (latency; the time interval between stimulus and response) were measured.

### Statistical Analysis

All statistical analyses were performed using Statistical Package for the Social Sciences (SPSS, ver: 19.0), and the comparisons of the means of the physiological parameters were conducted using non-parametric Kruskal Wallis and Mann-Whitney tests. The statistical significance was considered as P<0.05. 

## Results

### Muscle Stimulation

**Figure 1:**
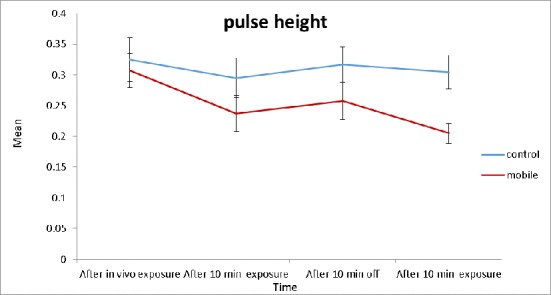
Pulse height of contraction (ph) – Muscle Stimulation (Error bars indicate SE)

**Figure 2:**
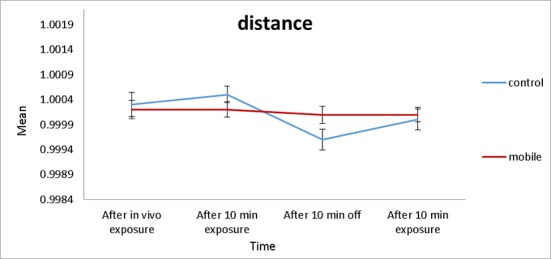
The time interval between two subsequent contractions (distance) – Muscle Stimulation (Error bars indicate SE)

**Figure 3:**
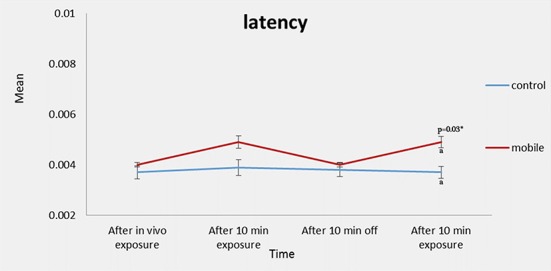
The latency period – Muscle Stimulation (Error bars indicate SE)

Table 1 shows the pulse height of contractions, the time interval between two subsequent contractions and the latency period in non-exposed control group after in-vivo sham-exposure, and when isolated gastrocnemius muscles were sham-exposed to mobile phone radiation. As it was expected, no statistically significant differences were observed in different sham exposure phases. The pulse height of contractions, the time interval between two subsequent contractions and the latency period in mobile phone group after in-vivo exposure, and when isolated gastrocnemius muscles were exposed to GSM mobile phone radiation are summarized in Table 2. As it is indicated in the table, there is a statistically significant difference among the latency periods in different off/on exposure phases (P=0.003). The mean (±SD) latency period after a 10 min in-vivo exposure was 0.004 ± 0.000 seconds but when isolated gastrocnemius muscles were exposed to mobile phone radiation it increased to 0.005 ± 0.001 seconds. Then, the latencies after 10 min off phase and 10 min exposure phase were 0.004 ± 0.000 and 0.005 ± 0.000 seconds, respectively. The inter-group comparison could not show any statistically significant differences in pulse height and time interval between two subsequent contractions (Figure 1-2). However, a statically significant difference (P=0.03) was found in the latency (Figure 3).

**Table 1:** The pulse height of contractions, the time interval between two subsequent contractions and the latency period in non-exposed control group after in-vivo sham-exposure, and when isolated gastrocnemius muscles were sham-exposed to mobile phone radiation (muscle stimulation).

**Control**	**Sample size**	**Phase I**	**Phase II**	**Phase III**	**Phase IV**	**p-value**
		After in vivo sham-exposure	After 10 min sham-exposure	After 10 min off	After 10 min sham-exposure	
Pulse height of contraction(mV)	10	0.324 ± 0.180	0.295 ± 0.163	0.316 ± 0.147	0.304 ± 0.140	0.68
Time interval(sec)	10	1 ± 0.001	1 ± 0.0008	0.999 ± 0.001	1 ± 0.001	0.083
Latency period(sec)	10	0.004 ± 0.001	0.004 ± 0.002	0.004 ± 0.001	0.004 ± 0.001	0.79

**Table 2:** **Panel A:** The pulse height of contractions, the time interval between two subsequent contractions and the latency period in mobile phone group after in-vivo exposure, and when isolated gastrocnemius muscles were exposed to GSM mobile phone radiation (muscle stimulation). **Panel B:** Pairwise comparisons of the latency period.

**Panel A**									
**Mobile Phone**	**Sample size**	**Phase I**		**Phase II**		**Phase III**		**Phase IV**	**p-value**
		After in vivo exposure		After 10 min exposure		After 10 min off		After 10 min exposure	
Pulse height of contraction(mV)	10	0.307 ± 0.139		0.237 ± 0.147		0.257 ± 0.154		0.205 ± 0.083	0.086
Time interval(sec)	10	1 ± 0.0009		1 ± 0.0007		1 ± 0.0008		1 ± 0.000	0.53
Latency period(sec)	10	0.004 ± 0.000		0.005 ± 0.001		0.004 ± 0.000		0.005 ± 0.000	0.003
**Panel B**									
**Latency period**	**Phase I**	Phase II			0.032		
		Phase III			0.2		
		Phase IV			0.02		
	**Phase II**	Phase III			0.018		
		Phase IV			0.018		
	**Phase III**	Phase IV			0.018		

### Nerve Stimulation

Table 3 shows the pulse height of contractions, the time interval between two subsequent contractions and the latency period in non-exposed control group after in-vivo sham-exposure, and when isolated gastrocnemius muscles were sham-exposed to mobile phone radiation. In contrast with what was expected, a statistically significant difference was observed for the pulse height of contractions in different sham exposure phases(P=0.033).

**Table 3:** **Panel A:** The pulse height of contractions, the time interval between two subsequent contractions and the latency period in non-exposed control group after in-vivo sham-exposure, and when isolated gastrocnemius muscles were sham-exposed to mobile phone radiation (Nerve stimulation). **Panel B:** Pairwise comparisons of the pulse height of contractions.

**Panel A**									
**Control**	**Sample size**	**Phase I**		**Phase II**		**Phase III**		**Phase IV**	**p-value**
		After in vivo sham-exposure		After 10 min sham-exposure		After 10 min off		After 10 min sham-exposure	
Pulse height of contraction(mV)	10	0.447 ± 0.18		0.424 ± 0.187		0.368 ± 0.189		0.353 ± 0.208	0.033
Time interval(sec)	10	0.999 ± 0.001		1 ± 0.0009		1 ± 0.001		1 ± 0.001	0.69
Latency period(sec)	10	0.004 ± 0.001		0.005 ± 0.001		0.005 ± 0.001		0.005 ± 0.001	0.093
**Panel B**									
**Pulse height of contraction**	**Phase I**	Phase II		0.88		
		Phase III		0.12		
		Phase IV		0.12		
	**Phase II**	Phase III		0.09		
		Phase IV		0.04		
	**Phase III**	Phase IV		0.39		

The pulse height of contractions, the time interval between two subsequent contractions and the latency period in mobile phone group after in-vivo exposure, and when isolated gastrocnemius muscles were exposed to GSM mobile phone radiation are summarized in Table 4. As it is indicated in this table, in contrast with muscle stimulation, there is a statistically significant difference among the pulse height of contractions and the latency periods in different off/on exposure phases (P=0.006 and P=0.001, respectively). The mean (±SD) pulse height of contractions after a in-vivo exposure was 0.312 ± 0.189 mV but when isolated gastrocnemius muscles were exposed to mobile phone radiation, these values for 10 minutes of on/off/on exposure phases were 0.173 ± 0.185, 0.180 ± 0.151 and 0.131 ± 0.112 mV, respectively. On the other hand, the mean (±SD) latency period after a 10 min in-vivo exposure was 0.005 ± 0.001 seconds but when isolated gastrocnemius muscles were exposed to mobile phone radiation it increased to 0.007 ± 0.001 seconds. Then, the latencies after 10 min off phase and 10 min exposure phase were 0.006 ± 0.001 and 0.007 ± 0.001 seconds, respectively.

**Table 4:** **Panel A:** The pulse height of contractions, the time interval between two subsequent contractions and the latency period in mobile phone group after in-vivo exposure, and when isolated gastrocnemius muscles were exposed to GSM mobile phone radiation (Nerve stimulation). **Panel B:** Pairwise comparisons of the pulse height of contractions. Panel C: Pairwise comparisons of the Latency period.

**Panel A**									
**Mobile Phone**	**Sample size**	**Phase I**		**Phase II**		**Phase III**		**Phase IV**	**p-value**
		After in vivo exposure		After 10 min exposure		After 10 min off		After 10 min exposure	
Pulse height of contraction(mV)	10	0.312 ± 0.189		0.173 ± 0.185		0.180 ± 0.151		0.131± 0.112	0.006
Time interval(sec)	10	1 ± 0.0007		1 ± 0.0006		1 ± 0.0009		1 ± 0.001	0.62
Latency period(sec)	10	0.005 ± 0.001		0.007± 0.001		0.006± 0.001		0.007 ± 0.001	0.001
**Panel B**									
**Pulse height of contraction**	**Phase I**	Phase II		0.04		
		Phase III		0.03		
		Phase IV		0.02		
	**Phase II**	Phase III		0.80		
		Phase IV		0.68		
	**Phase III**	Phase IV		0.17		
**Panel C **									
**Latency period **	**Phase I**	Phase II		0.11		
		Phase III		0.23		
		Phase IV		0.04		
	**Phase II**	Phase III		0.13		
		Phase IV		0.38		
	**Phase III**	Phase IV		0.02		

**Figure 4:**
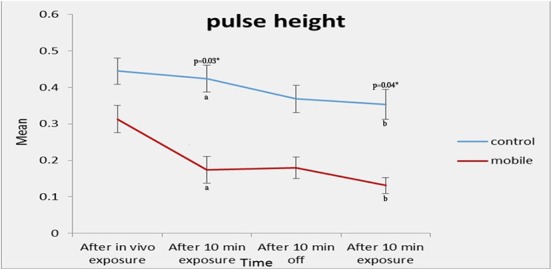
Pulse height of contraction (ph) – Nerve Stimulation (Error bars indicate SE)

**Figure 5:**
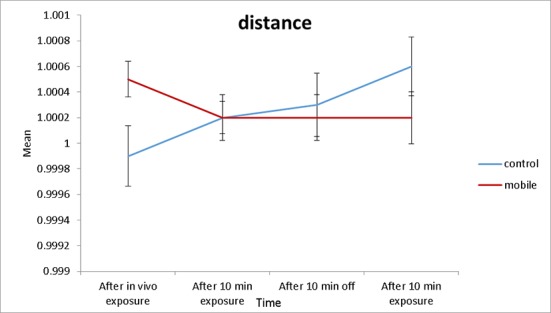
The time interval between two subsequent contractions – Nerve Stimulation (Error bars indicate SE)

**Figure 6:**
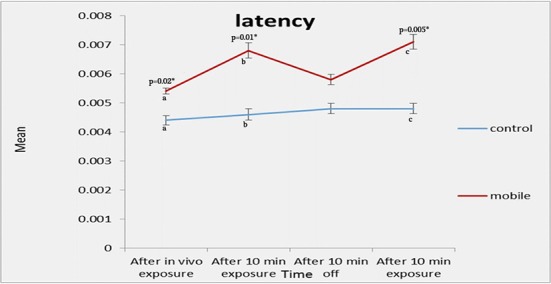
The time interval between two subsequent contractions – Nerve Stimulation (Error bars indicate SE)

The inter-group comparison showed statistically significant differences in pulse height (Figure 4). However, we could not show any statically significant difference in the time interval between two subsequent contractions (Figure 5). On the other hand, statically significant differences  were found in the latency (Figure 6).

Table 5 shows the calculated SARs for frequencies of 900 and 1800 MHz at distances of 10 and 15 cm from the source.

**Table 5:** Calculated SARs for frequencies of 900 and 1800 MHz at distances of 10 and 15 cm from the source.

**Distance(cm)**	**Frequency(MHZ)**	**SAR (muscle)**	**SAR (nerve)**
15	900	0.669 w/kg	0.407 w/kg
15	1800	0.231 w/kg	0.145 w/kg
10	900	1.4 w/kg	0.909 w/kg
10	1800	0.51 w/kg	0.32 w/kg

## Discussion

Findings of this study show that the pulse height of contractions in frog’s gastrocnemius muscle can be affected by the exposure to electromagnetic fields. Especially, our experiments showed that the latency period can be easily altered by the irradiation. However, none of the experiments could show an alteration in the time interval between two subsequent contractions after exposure to electromagnetic fields.  The general pattern which could be observed in these experiments indicates that exposure to electromagnetic fields usually increase the latency period. To the best of our knowledge, this is the first investigation that reports the detrimental biological effects of exposure to mobile phone radiation on the pulse height of contractions, time interval between two subsequent contractions and the latency period in frog’s gastrocnemius muscle after applying single square pulses of 1V (1 Hz) as stimuli.

In general terms, our findings are in agreement with published reports which showed that many cells such as epithelial, endothelial and epidermal cells, cardiac muscle cells, fibroblasts as well as yeast, E. coli, developing chick eggs, and dipteran cells respond to EMF, both in vivo and in vitro [30]. Tissue cultured cells have been reported to be less susceptible to the effects of EMF. This fall in the susceptibility to the effects of EMF can possibly be due to this fact that immortalized cells can be altered in an important manner to be able to live limitlessly in unnatural laboratory conditions. However, our findings cannot confirm the results obtained in the studies which indicated that exposure of the isolated frog sciatic nerves, cat saphenous nerves, rabbit vagus nerves, superior cervical ganglia, and rat diaphragm muscles to 2450 MHz microwave radiation does not alter the attribute of nerves or muscles exposed to continuous wave (CW) radiation with specific absorption rate (SAR) of 0.3-1500 W/kg and pulsed peak SAR of 0.3-220 kW/kg [31].

On the other hand, these findings generally support the early reports which revealed the non-thermal effects of electromagnetic radiation on amphibians [32, 33]. It is worth mentioning that some early reports have shown teratogenic effects in amphibians exposed to electromagnetic fields [34]. Furthermore, it has been shown that common frogs (Rana temporaria L.) developed under exposure to electromagnetic field exhibit a higher rate of mortality, slow and less synchronously development, allergies, and alterations in their blood counts [35]. It has also been reported that frogs exposed to electromagnetic radiation with power densities of 30–60 mWcm-2 show heart rhythm change, possibly due to activation of the nervous system. Moreover, a rise in the heart rate and arrhythmia after exposure of toad hearts to pulses of 1425 MHz at a power density of 0.6 mWcm-2, has been reported [32]. In Spain, Balmori reported a possible association of exposure to microwave radiation and the global disappearance of frogs [36].

## Conclusion

Findings of this study clearly showed that the pulse height of contractions muscle could be affected by the exposure to mobile phone radiation. Moreover, the latency period was effectively altered in RF-exposed samples. However, none of our experiments could show an alteration in the time interval between two subsequent contractions after exposure to mobile phone radiation. Altogether, these findings support the early reports which showed a wide variety of non-thermal effects of electromagnetic radiation on amphibians including the effects on the pattern of muscle extractions. 

## References

[1] Haarala C, Bergman M, Laine M, Revonsuo A, Koivisto M, Hamalainen H (2005). Electromagnetic field emitted by 902 MHz mobile phones shows no effects on children’s cognitive function. *Bioelectromagnetics*.

[2] Eulitz C, Ullsperger P, Freude G, Elbert T (1998). Mobile phones modulate response patterns of human brain activity. *Neuroreport*.

[3] Freude G, Ullsperger P, Eggert S, Ruppe I (1998). Effects of microwaves emitted by cellular phones on human slow brain potentials. *Bioelectromagnetics*.

[4] Urban P, Lukas E, Roth Z (
1998
). Does acute exposure to the electromagnetic field emitted by a mobile phone influence visual evoked potentials?. A pilot study. *Cent Eur J Public Health*.

[5] Hladky A, Musil J, Roth Z, Urban P, Blazkova V (1999). Acute effects of using a mobile phone on CNS functions. *Cent Eur J Public Health*.

[6] Freude G, Ullsperger P, Eggert S, Ruppe I (2000). Microwaves emitted by cellular telephones affect human slow brain potentials. *Eur J Appl Physiol*.

[7] Jech R, Sonka K, Ruzicka E, Nebuzelsky A, Bohm J, Juklickova M (2001). Electromagnetic field of mobile phones affects visual event related potential in patients with narcolepsy. *Bioelectromagnetics*.

[8] Aalto S, Haarala C, Brück A, Sipilä H, Hämäläinen H, Rinne JO (2006). Mobile phone affects cerebral blood flow in humans. *Journal of Cerebral Blood Flow & Metabolism*.

[9] Hamblin DL, Wood AW, Croft RJ, Stough C (2004). Examining the effects of electromagnetic fields emitted by GSM mobile phones on human event-related potentials and performance during an auditory task. *Clin Neurophysiol*.

[10] Hinrichs H, Heinze HJ (2004). Effects of GSM electromagnetic field on the MEG during an encoding-retrieval task. *Neuroreport*.

[11] Krause CM, Haarala C, Sillanmaki L, Koivisto M, Alanko K, Revonsuo A (2004). Effects of electromagnetic field emitted by cellular phones on the EEG during an auditory memory task: a double blind replication study. *Bioelectromagnetics*.

[12] Hamblin DL, Croft RJ, Wood AW, Stough C, Spong J (2006). The sensitivity of human event-related potentials and reaction time to mobile phone emitted electromagnetic fields. *Bioelectromagnetics*.

[13] Ferreri F, Curcio G, Pasqualetti P, De Gennaro, Fini R, Rossini PM (2006). Mobile phone emissions and human brain excitability. *Ann Neurol*.

[14] Papageorgiou CC, Nanou ED, Tsiafakis VG, Kapareliotis E, Kontoangelos KA, Capsalis CN (2006). Acute mobile phone effects on pre-attentive operation. *Neurosci Lett*.

[15] Yuasa K, Arai N, Okabe S, Tarusawa Y, Nojima T, Hanajima R (2006). Effects of thirty minutes mobile phone use on the human sensory cortex. *Clin Neurophysiol*.

[16] Mortazavi SM, Motamedifar M, Namdari G, Taheri M, Mortazavi AR, Shokrpour N (2014). Non-linear adaptive phenomena which decrease the risk of infection after pre-exposure to radiofrequency radiation. *Dose Response*.

[17] Mortazavi SM, Taeb S, Dehghan N (2013). Alterations of visual reaction time and short term memory in military radar personnel. *Iran J Public Health*.

[18] Mortazavi SM, Rouintan MS, Taeb S, Dehghan N, Ghaffarpanah AA, Sadeghi Z (2012). Human short-term exposure to electromagnetic fields emitted by mobile phones decreases computer-assisted visual reaction time. *Acta Neurol Belg*.

[19] Mortazavi S, Mosleh-Shirazi M, Tavassoli A, Taheri M, Mehdizadeh A, Namazi S (2013). Increased Radioresistance to Lethal Doses of Gamma Rays in Mice and Rats after Exposure to Microwave Radiation Emitted by a GSM Mobile Phone Simulator. *Dose Response*.

[20] Mortazavi S, Mosleh-Shirazi M, Tavassoli A, Taheri M, Bagheri Z, Ghalandari R (2011). A comparative study on the increased radioresistance to lethal doses of gamma rays after exposure to microwave radiation and oral intake of flaxseed oil. *Iranian Journal of Radiation Research*.

[21] Mortavazi S, Habib A, Ganj-Karami A, Samimi-Doost R, Pour-Abedi A, Babaie A (2009). Alterations in TSH and Thyroid Hormones following Mobile Phone Use. *Oman Med J*.

[22] Mortazavi SM, Daiee E, Yazdi A, Khiabani K, Kavousi A, Vazirinejad R (2008). Mercury release from dental amalgam restorations after magnetic resonance imaging and following mobile phone use. *Pak J Biol Sci*.

[23] Mortazavi SM, Ahmadi J, Shariati M (2007). Prevalence of subjective poor health symptoms associated with exposure to electromagnetic fields among university students. *Bioelectromagnetics*.

[24] Mortazavi S (2013). Safety issue of mobile phone base stations. *J Biomed Phys Eng*.

[25] Mortazavi SMJ (2004). Adaptive responses after exposure to cosmic and natural terrestrial radiation. *Indian Journal of Radiation Research*.

[26] Mortazavi SMJ, Tavassoli A, Ranjbari F, Moammaiee P (2010). Effects of laptop computers’ electromagnetic field on sperm quality. *Journal of Reproduction & Infertility*.

[27] Mortazavi SM, Vazife-Doost S, Yaghooti M, Mehdizadeh S, Rajaie-Far A (2012). Occupational exposure of dentists to electromagnetic fields produced by magnetostrictive cavitrons alters the serum cortisol level. *J Nat Sci Biol Med*.

[28] Mortazavi SM, Daiee E, Yazdi A, Khiabani K, Kavousi A, Vazirinejad R (2008). Mercury release from dental amalgam restorations after magnetic resonance imaging and following mobile phone use. *Pak J Biol Sci*.

[29] Mortazavi SM, Neghab M, Anoosheh SM, Bahaeddini N, Mortazavi G, Neghab P (2014). High-field MRI and mercury release from dental amalgam fillings. *Int J Occup Environ Med*.

[30] Blank M, Goodman R (2009). Electromagnetic fields stress living cells. *Pathophysiology*.

[31] Chou C-k, Guy AW (1978). Effects of electromagnetic fields on isolated nerve and muscle preparations. *Microwave Theory and Techniques, IEEE Transactions on*.

[32] Frey AH, Seifert E (1968). Pulse modulated UHF energy illumination of the heart associated with change in heart rate. *Life Sciences*.

[33] Neurath PW (1968). High gradient magnetic field inhibits embryonic development of frogs. *Nature*.

[34] Levengood WC (1969). A new teratogenic agent applied to amphibian embryos. *J Embryol Exp Morphol*.

[35] Grefner N, Yakovleva T, Boreisha I (
1998
). Effects of electromagnetic radiation on tadpole development in the common frog (Rana temporaria L.). *Russian journal of ecology*.

[36] Balmori A (2006). The incidence of electromagnetic pollution on the amphibian decline: Is this an important piece of the puzzle?. *Toxicological & Environmental Chemistry*.

